# The *Non-Coding RNA* Journal Club: Highlights on Recent Papers—9

**DOI:** 10.3390/ncrna7030058

**Published:** 2021-09-16

**Authors:** Neil Renwick, Assam El-Osta, Irene Salamon, Elisabetta Broseghini, Manuela Ferracin, Laura Poliseno, Stanislovas S. Jankauskas, Gaetano Santulli, Hua Xiao, Patrick K. T. Shiu, Souvick Roy, Ajay Goel

**Affiliations:** 1 Laboratory of Translational RNA Biology, Department of Pathology and Molecular Medicine, Queen’s University, Kingston, ON K7L 3N6, Canada; 2 Department of Diabetes, Central Clinical School, Monash University, Melbourne, VIC 3004, Australia; 3 Epigenetics in Human Health and Disease Laboratory, Central Clinical School, Monash University, Melbourne, VIC 3004, Australia; 4 Department of Medicine and Therapeutics, The Chinese University of Hong Kong, Hong Kong, China; 5 Hong Kong Institute of Diabetes and Obesity, Prince of Wales Hospital, The Chinese University of Hong Kong, 3/F Lui Che Woo Clinical Sciences Building, 30-32 Ngan Shing Street, Sha Tin, Hong Kong, China; 6 Li Ka Shing Institute of Health Sciences, The Chinese University of Hong Kong, Hong Kong, China; 7 Biomedical Laboratory Science, Department of Technology, Faculty of Health, University College Copenhagen, 1799 Copenhagen, Denmark; 8 Department of Experimental, Diagnostic and Specialty Medicine, University of Bologna, 40126 Bologna, Italy; irene.salamon@unibo.it (I.S.); elisabett.broseghin2@unibo.it (E.B.); 9 Institute of Clinical Physiology, CNR, 56124 Pisa, Italy; 10 Oncogenomics Unit, Core Research Laboratory, ISPRO, 56124 Pisa, Italy; 11 Albert Einstein College of Medicine, Wilf Family Cardiovascular Research Center, New York City, NY 10461, USA; stanislovas.jankauskas@einsteinmed.org; 12 Division of Biological Sciences, University of Missouri, Columbia, MO 65211, USA; xiaohu@missouri.edu; 13 Department of Molecular Diagnostics and Experimental Therapeutics, Beckman Research Institute of City of Hope, Biomedical Research Center, Monrovia, CA 91016, USA; soroy@coh.org; 14 City of Hope Comprehensive Cancer Center, Duarte, CA 91010, USA

## 1. Introduction

We are delighted to share with you our ninth Journal Club and highlight some of the most interesting papers published recently. We hope to keep you up-to-date with non-coding RNA research works that are outside your study area. The *Non-Coding RNA* Scientific Board wishes you an exciting and fruitful read.

## 2. Small RNAs Are Modified with N-glycans and Displayed on the Surface of Living Cells

### Highlight by Neil Renwick 

Mammalian glycans are short sugar chains that regulate cellular processes through macromolecular attachment. Although lipids and proteins are well-known targets of glycosylation, RNA is not thought to be a scaffold for glycan modification. In a recent issue of *Cell*, Carolyn Bertozzi’s group reports on glycoRNAs—glycan-RNA conjugates that reside on the cell surface and interact with anti-dsRNA antibodies and proteins that bind sialic acid. 

Leveraging expertise in metabolic labeling and biorthogonal chemistry, genetics, and biochemistry, the authors discovered and characterized glycoRNAs in multiple mammalian cell lines and tissues [1]. Despite migrating as high molecular weight (>10 kb) species in denaturing agarose gels, glycoRNAs were found to comprise small non-coding RNAs with highly sialylated and fucosylated N-glycan modifications. These molecules were also found mostly on cell surfaces where they can interact with anti-dsRNA antibodies and bind to members of the sialic acid-binding immunoglobulin-like lectin (Siglec) receptor family.

Although more work is needed to define the range of glycan modifications, determine the precise nature of the glycan-RNA linkage, delineate glycoRNA biosynthesis and trafficking mechanisms, elucidate roles in extracellular biology, and explore the implications for autoimmune and other diseases, this landmark paper “points to a new axis of RNA glycobiology” [1].

## 3. Biomarkers of Broken Heart Syndrome

### Highlight by Assam El-Osta

Takotsubo cardiomyopathy, also known as broken heart syndrome, is characterized by transient systolic dysfunction of the left ventricle not attributed to coronary artery disease. This condition follows experiences of extreme emotional or physical stress and is more common in women. While cardiologists have known for some time that broken heart syndrome exists, this condition remains poorly understood. Previously shown to be elevated in Takotsubo patients, a recent study assessed miR-16 and miR-26a in a pre-clinical model of Takotsubo-like cardiomyopathy using subcutaneous adrenaline injection [2]. In a rat model, serum production and release of miR-16 and miR-26a was unaltered following stimulation by adrenaline, suggesting that the catecholamine surge does not coordinate miRNA regulation. As a resolution to this conundrum, the authors postulated that the elevated miR-16 and miR-26a in human Takotsubo patients described in previous studies may precede the catecholamine rise induced by adrenaline, as observed in their studies. Surprisingly, they discovered advanced adrenaline-induced Takotsubo-like injury when cardiac AAV miR16 and miR26a were co-expressed in rats. Corresponding transfection studies using cardiomyocytes isolated from adult rats emphasized that the miRs reduced baseline contractility by transfection. Closely associated with reduced calcium cycling by miR16 and miR26a, the authors observed similar results using non-failing human cardiomyocytes. The true physiological targets of miR16 and miR26a were questioned and studies confirmed reduced CACNB1, RGS4, and GNB1 expression. Taken together, the pre-clinical findings implicate miR-16 and miR-26a as biomarkers and signaling determinants for susceptibility to broken heart syndrome. Future studies examining the origins of Takotsubo by studying the brain—heart axis and how miRNAs serve as nodes interacting with autonomic signaling networks are warranted.

## 4. The Battle of Nascent RNAs for Chromatin Regulation

### Highlight by Irene Salamon, Elisabetta Broseghini and Manuela Ferracin

Non-coding RNAs and enhancer RNAs control protein—chromatin interactions at specific sites through the binding of transcription factors and chromatin remodeling proteins. In a recent paper published in *Molecular Cell*, Skalska at al. described how nascent RNA binds a set of proteins, thus hindering their activity on chromatin [3].

The authors observed that the inhibition of RNA Pol II transcription and induction of RNA degradation in mouse ESC produced a wide effect on protein—chromatin interactions: a set of proteins became unbound from chromatin, and another group enriched on chromatin. Moreover, they described how this control takes place, demonstrating that nuclear RNAs affect the direct interaction of proteins with nucleosomes and, furthermore, that the antagonistic action of RNA is due to a physical bond between proteins and RNA. As an example of a generalized mechanism, they described the regulation of P-TEFb (a transcriptional elongation factor), which was one of the most enriched chromatin-associated proteins upon RNA Pol II inhibition. P-TEFb binds nascent pre-mRNAs and is regulated by 7SK ribonucleoprotein. 

These findings demonstrate a novel and unexpected role of nuclear transcripts as antagonists of interactions between regulatory proteins and chromatin. Further studies are needed to identify the chromatin sites affected by this specific RNA regulation and how RNA contributes to the regulation of chromatin state.

## 5. No Co-expression, No Sponging

### Highlight by Laura Poliseno

In their article “*Spatial expression analyses of the putative oncogene ciRS-7 in cancer reshape the microRNA sponge theory*” [4], Kristensen and colleagues meritoriously point out that correlation data can support competing endogenous RNA (ceRNA)-based interactions only if ceRNA partners, and the microRNA they compete for, are co-expressed in the same cell type. 

When it comes to explaining the oncogenic features of ciRS-7 circRNA, the extremely high number of binding sites for oncosuppressive miR-7 is a characteristic that calls for sponging. However, by employing multiple techniques that allow them to spatially resolve expression patterns, the authors establish that, in colon cancer, ciRS-7 is expressed only in stromal cells, while miR-7 is expressed only in cancer cells. If this was not enough to kill the hypothesis that, in this specific context, ciRS-7 works as a sponge for miR-7, they also show that the positive correlation that is observed between ciRS-7 and putative miR-7 target genes—and that is often taken as indicative of ceRNA-based interaction—is rather ascribable to stromal co-expression and in fact applies also to other stromal circRNAs that do not contain miR-7 binding sites. 

This work elegantly reminds us that ceRNA-based interactions cannot rely only on correlation, while appropriate gain and loss-of-function experiments are crucial to seal the deal. It also prompts the thorough investigation of co-expression, which should be valued as the prerequisite for a relevant correlation.

## 6. Tackling SARS-CoV-2 Variants Using Specific miRNA Tools

### Highlight by Stanislovas S. Jankauskas and Gaetano Santulli

Effective therapeutic strategies against the coronavirus disease 2019 (COVID-19) caused by the severe acute respiratory syndrome coronavirus 2 (SARS-CoV-2) are desperately needed. In a recent issue of *Frontiers in Genetics*, Chiara Siniscalchi and collaborators [5] identify some miRNAs targeting SARS-CoV-2 RNA. Specifically, starting with a well-designed bioinformatic analysis that pinpointed eight candidate miRNAs, the authors went on to validate the functional interaction of five miRNAs (namely miR-15b-5p, miR-29a-3p, miR-30c-5p, miR-219a-2-3p, and miR-378d) with viral sequences in a human lung cell line. 

Of interest, the viral target sequences are fully conserved in more recent variants of SARS-CoV-2. Moreover, the authors elegantly prove that miR-15b is able to repress plasmid-driven spike expression.

This discovery could have major implications in clinical scenarios, since inhibiting the expression of the spike transcript and targeting viral sequences of SARS-CoV-2 may provide unprecedented therapeutic approaches for the treatment of COVID-19.

## 7. A Truncated Dicer Mediates Antiviral RNA Interference (RNAi) in Mammalian Stem Cells

### Highlight by Hua Xiao and Patrick K. T. Shiu

In mammals, interferon (IFN) signaling pathways are the first line of defense against viral infections for differentiated cells. Stem cells, on the other hand, are refractory to IFN induction and signaling, and they instead utilize constitutively expressed (IFN-independent) restriction factors for protection. Invertebrates and plants do not have an IFN system, and they combat viruses using RNAi. Antiviral RNAi begins with Dicer, which cleaves viral double-stranded RNA (dsRNA) replicative intermediates into small interfering RNAs (siRNAs). These siRNAs subsequently guide the slicing of viral RNAs. In a recent article of *Science*, Poirier and others reported that mammalian stem cells are also able to harness RNAi for antiviral immunity [6].

Mammals contain only one *DICER* gene, whose canonical protein cuts precursor microRNAs (pre-miRNAs) well but processes dsRNAs poorly. In this study, the authors discovered an alternatively spliced isoform of Dicer called antiviral Dicer (aviD) in stem cells. The *aviD* transcript lacks exons 7 and 8, resulting in the deletion of a helicase subdomain that negatively regulates Dicer’s ability to process dsRNAs. Accordingly, aviD can effectively dice viral dsRNAs into siRNAs and curb viral infections in stem cells. This antiviral RNAi system does not appear to restrict the replication of DNA viruses, presumably because only RNA viruses can produce a significant amount of dsRNAs.

It is interesting that different defense strategies are deployed for different cell differentiation statuses. Perhaps mammals have evolved to protect their precious stem cells from the toxic effects of IFN exposure. The scientists here have demonstrated the effectiveness of antiviral RNAi against Zika and COVID-19 viruses. Future research in this area will undoubtedly expedite the improvement of antiviral therapeutics [7].

## 8. CircNEIL3—A Prognostic Biomarker and Potential Therapeutic Target in Pancreatic Ductal Adenocarcinoma

### Highlight by Souvick Roy and Ajay Goel

Pancreatic ductal adenocarcinoma (PDAC) is a highly aggressive malignancy and is estimated to become the second leading cause of cancer-related deaths by 2030. Surgical resection for localized PDAC is considered the only curative treatment option for long-term survival; however, more than 80% of patients at initial diagnosis have an unresectable or borderline resectable disease, limiting their chances to receive a curative treatment. Unfortunately, even in PDAC patients who undergo surgical resection, the 5-year survival rates are quite poor, ranging between 10% and 25%, primarily due to the local and distant metastasis of disease. These findings highlight the unmet clinical need to identify robust prognostic biomarkers, as well as potential therapeutic targets, which can help improve overall survival outcomes in patients suffering from PDAC. In a recent study published by Shen and colleagues, the authors report exciting novel findings, which reveal that the regulatory loop of circNEIL3 facilitates proliferation and metastasis in PDAC, that the expression levels of this circRNA can be used as a prognostic marker, and that this non-coding RNA may also serve as a potential therapeutic target in PDAC [8].

In this elegant article, the authors identified that increased expression of circNEIL3 in PDAC tumor tissues was an independent risk factor associated with poor prognosis. Subsequent in vitro and in vivo functional experiments supported the oncogenic role of circNEIL3 through its role as a sponge to inhibit the tumor suppressive activity of miR-432-5p. In addition, this functional role of circNEIL3 subsequently promoted A-to-I RNA editing by regulating the expression of ADAR1. The gene regulatory activity of circNEIL3 was reported to be manifested through a feedback loop of the circNEIL3/miR-432-5p/ADAR1/GLI axis, by regulating cell cycle and epithelial-mesenchymal transition (EMT) pathways. These results are quite fascinating and provide novel evidence for a circRNA to serve as a prognostic biomarker, with a potential for therapeutic targeting in this malignancy—opening up a new concept for research and an important step forward as we usher into the era of precision oncology.

## References

[1] Flynn R.A., Pedram K., Malaker S.A., Batista P.J., Smith B.A.H., Johnson A.G., George B.M., Majzoub K., Villalta P.W., Carette J.E. (2021). Small RNAs are modified with N-glycans and displayed on the surface of living cells. Cell.

[2] Couch L.S., Fiedler J., Chick G., Clayton R., Dries E., Wienecke L.M., Fu L., Fourre J., Pandey P., Derda A.A. (2021). Circulating microRNAs predispose to takotsubo syndrome following high-dose adrenaline exposure. Cardiovasc. Res..

[3] Skalska L., Begley V., Beltran M., Lukauskas S., Khandelwal G., Faull P., Bhamra A., Tavares M., Wellman R., Tvardovskiy A. (2021). Nascent RNA antagonizes the interaction of a set of regulatory proteins with chromatin. Mol. Cell.

[4] Kristensen L.S., Ebbesen K.K., Sokol M., Jakobsen T., Korsgaard U., Eriksen A.C., Hansen T.B., Kjems J., Hager H. (2020). Spatial expression analyses of the putative oncogene ciRS-7 in cancer reshape the microRNA sponge theory. Nat. Commun..

[5] Siniscalchi C., Di Palo A., Russo A., Potenza N. (2021). Human Micrornas Interacting with Sars-Cov-2 Rna Sequences: Computational Analysis and Experimental Target Validation. Front. Genet..

[6] Poirier E.Z., Buck M.D., Chakravarty P., Carvalho J., Frederico B., Cardoso A., Healy L., Ulferts R., Beale R., Reis e Sousa C. (2021). An isoform of Dicer protects mammalian stem cells against multiple RNA viruses. Science.

[7] Shahrudin S., Ding S.W. (2021). Boosting stem cell immunity to viruses. Science.

[8] Shen P., Yang T., Chen Q., Yuan H., Wu P., Cai B., Meng L., Huang X., Liu J., Zhang Y. (2021). CircNEIL3 regulatory loop promotes pancreatic ductal adenocarcinoma progression via miRNA sponging and A-to-I RNA-editing. Mol. Cancer.

